# Degradation Induced by Total Ionizing Dose and Hot Carrier Injection in SOI FinFET Devices

**DOI:** 10.3390/mi15081026

**Published:** 2024-08-11

**Authors:** Hao Yu, Wei Zhou, Hongxia Liu, Shulong Wang, Shupeng Chen, Chang Liu

**Affiliations:** 1 Key Laboratory for Wide Band Gap Semiconductor Materials and Devices of Education Ministry, School of Microelectronics, Xidian University, Xi’an 710071, China; a13204462468@163.com (H.Y.); zhou_wei_fs@163.com (W.Z.); slwang@xidian.edu.cn (S.W.); spchen@xidian.edu.cn (S.C.); xd_liuchang@163.com (C.L.); 2 Science and Technology on Reliability Physics and Application Technology of Electronic Component Laboratory, China Electronic Product Relibility and Environmental Testing Research Institute, Guangzhou 511370, China

**Keywords:** SOI FinFET, TID, HCI, coupling effect, temperature, TCAD simulation

## Abstract

The working environment of electronic devices in the aerospace field is harsh. In order to ensure the reliable application of the SOI FinFET, the total ionizing dose (TID) and hot carrier injecting (HCI) reliability of an SOI FinFET were investigated in this study. First, the influence of TID on the device was simulated. The results show that TID causes the threshold voltage to decrease and the off-state current and subthreshold swing to increase. TID causes more damage to the device at high temperature and also reduces the saturation drain current of the device. HCI causes the device threshold voltage to increase and the saturation drain current to decrease. The HCI is more severe at high temperatures. Finally, the coupling effects of the two were simulated, and the results show that the two effects cancel each other out, and the degradation of various electrical characteristic parameters is different under different coupling modes.

## 1. Introduction

With the rapid development of the semiconductor manufacturing process, the transistor feature size continues to decrease, and the short-channel effect of the transistor plays a more significant role. Under this premise, FinFET and SOI devices were devised [1]. Compared with the traditional planar MOSFET, the channel of a tri-gate FinFET is wrapped by the gate, and the equivalent gate width is many times that of a MOSFET with the same channel width. Thus, the FinFET device has stronger gate control capability and better suppression of the short-channel effect [2], while the SOI device, with its full dielectric isolation structure, has obvious advantages in terms of anti-latchup and transient radiation capability [3]. The SOI FinFET has many of the advantages of both devices, making it a strong candidate for future aerospace and military electronics applications [4].

Due to the complex and harsh working environment in the aerospace and military defense fields, not only is the temperature span large, but a variety of space radiation and nuclear radiation types exist, so it is imperative to solve the problem of reliability of SOI FinFET devices in complex environments. This paper focuses on the total ionizing dose effect due to irradiation and the hot carrier injection damage caused by long-term device operation.

## 2. Device Simulation Modeling

In this study, we used the SDE tool of the Sentaurus TCAD simulation platform to build a 3D N-type SOI FinFET device model under a 14 nm process node, with the device structure parameters taken from IBM [4].

Firstly, the SDE tool was used to complete the construction of the 3D structure of the device, and the structural parameters of the device are shown in Table 1. Then, the device model was meshed using the Sentaurus Mesh tool. The three-dimensional device structure was compared to the two-dimensional structure of the overall complexity of the device to greatly increase the simulation calculations. Therefore, in the mesh processing for key areas such as the trench, the trench and gate oxide layer interface, the interface between the trench and the buried oxygen layer, and other areas with a smaller mesh size, the rest of the mesh was appropriately adjusted to reduce the number of simulation calculations.

**Figure 1:**
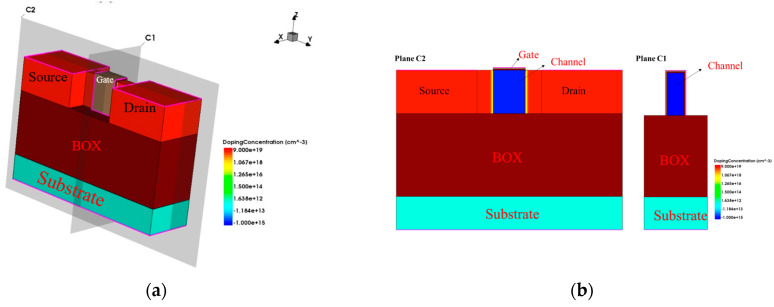
The 14 nm SOI FinFET device structure: (**a**) 3D schematic of SOI FinFET; (**b**) cross-sections of SOI FinFET.

Figure 1 shows the device structure, which is divided into four layers from the bottom to the top. The first layer is the silicon substrate material, providing support for the entire device structure; the second layer is the BOX buried oxygen layer, where the material type of the layer is silicon dioxide; the third layer is the channel, as well as the source and drain regions, where the highlighted channel is called the fins and the two ends of the channel are the source and drain; and the fourth layer is a very thin gate oxide layer composed of silicon dioxide and hafnium dioxide.

**Figure 2:**
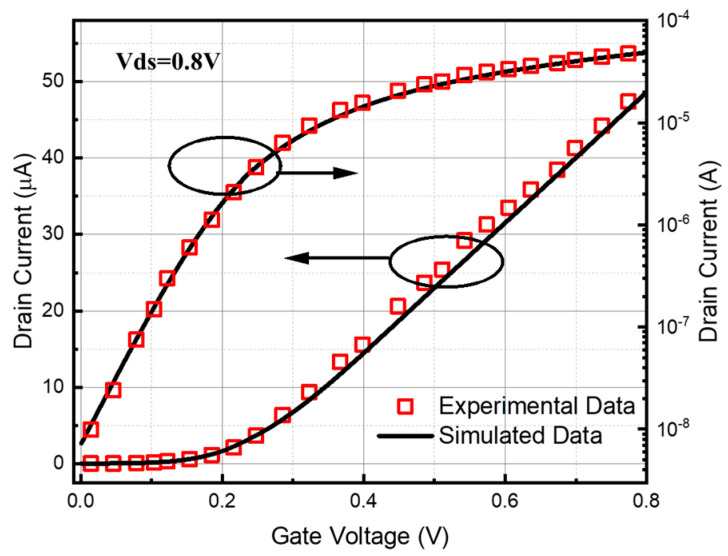
Comparison of transfer characteristics of drain current for the SOI FinFET with the experimental data [5].

The source-drain region of the device model is uniformly doped with a doping concentration of 4.5 × 10^19^ cm^−3^. The extended region of the source-drain portion in contact with the trench is Gaussian-doped, with a doping concentration of 1.0 × 10^15^ cm^−3^, to a depth of 2 nm. Afterwards, this paper compares the transfer characteristic curves of the references and the simulation with a Vds of 0.8 V; the calibrated comparison graph is shown in Figure 2.

## 3. TID and HCI Simulation

### 3.1. TID

#### 3.1.1. Mechanism and Physical Model of Action of TID

A silicon dioxide material in high-energy particle bombardment will produce electron-hole pairs. A portion of the electron-hole pairs will be quickly compounded in a short time, and the uncompounded electron–hole pairs will drift under the action of the electric field. Due to the difference in mobility, the electrons are quickly swept out of the oxide layer, and the holes with lower mobility slowly drift in the oxide layer. In this process, a portion of the holes are captured by the traps in the SiO_2_, which form a stable positively charged oxide trap charge, and a portion of the holes move to the interface, where they participate in the reaction to generate the interface state trap charge [6].

The physical model of total dose effect simulation includes the irradiation model, mobility model, generation recombination model, and thermodynamic dynamic model. The radiation model includes the radiation model and the traps model. “OxideAsSemiconductor” should be used to replace SiO_2_ when simulating the TID.

#### 3.1.2. Simulation of TID

**Figure 3:**
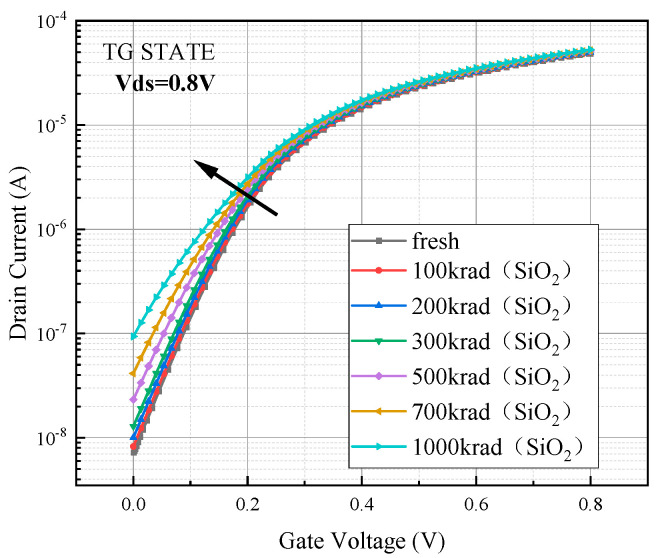
Degradation of device transfer characteristic curves after simulation of TID.

Based on the above device model, the degradation of the electrical parameters of the device during irradiation was simulated. During the simulation process, the device was in TG bias state (Vs = Vd = 0.8 V, Vg = 0 V), the simulation temperature was set to 300 K, the irradiation dose rate was 1 krad (SiO_2_)/s, and the irradiation doses were 100 krad (SiO_2_), 200 krad (SiO_2_), 300 krad (SiO_2_), 500 krad (SiO_2_), 700 krad (SiO_2_), and 1000 krad (SiO_2_). While electronic devices are commonly subjected to long-term irradiation conditions in many practical applications at low dose rates, typically ranging from a few hundred rad (SiO_2_)/s to a few rad (SiO_2_)/s, the use of a higher dose rate of 1 krad (SiO_2_)/s in this thesis expedites the observation of irradiation effects. This also expedites the research process by acquiring the potential impacts of long-term radiation on a material or technology in a shorter amount of time. Furthermore, high dose rates can also mimic how devices might function in harsh environments, such as space habitats or specific applications where they may be subjected to high dose rate irradiation conditions. The device transfer characteristic curves were extracted after the irradiation simulation was completed, Vds was set to 0.8 V, and the gate voltage was scanned from zero to 0.8 V. The degradation of the transfer characteristic curves shown in Figure 3 was obtained.

Figure 3 shows the degradation of the transfer characteristic curve of the device in logarithmic coordinates. From the above figure, it can be seen that the device transfer characteristic curve under the action of TID will drift upward as a whole, and the curvature of the line becomes straighter. The following Table 2 was obtained by extracting the threshold voltage, off-state current, subthreshold swing, and other parameters from the transfer characteristic curve.

After simulating the total dose effect, the device threshold voltage decreases and the off-state current and subthreshold swing increase. When the irradiation dose reaches 1 Mrad (SiO_2_), the threshold voltage degradation exceeds 10%, the subthreshold swing degradation is close to 40%, and the off-state current grows to 13 times the initial value.

The total threshold voltage displacement is the sum of the threshold voltage displacements due to the oxide layer trap charge and the interface state trap charge.

(1) $$\Delta V_{th}=\Delta V_{ot}+\Delta V_{it}$$

In Equation (1), ∆*V_ot_* is the threshold voltage drift due to the oxide layer trap charge and ∆*V_it_* is the threshold voltage drift due to the interface trap charge:

(2) $$\Delta V_{ot}=-\frac{Q_{ot}}{C_{ox}},\;\Delta V_{it}=\frac{Q_{it}}{C_{ox}}$$

where *Q_ot_* denotes the oxide layer trap charge density and *Q_it_* is the interface state charge density.

With the extension of irradiation time, the radiation-induced charge in the buried oxygen layer of the device increases, the electrons with higher mobility escape from the buried oxygen layer under the action of the electric field, and the holes with lower mobility are trapped by the trap to become the positively charged oxide trap charge. When no gate voltage is applied, some electrons in the channel will still accumulate on the lower surface of the channel under the attraction of the positive trap charge of the buried oxygen layer, forming a conductive channel, resulting in the increase in leakage current. This is equivalent to applying a small positive voltage to the gate in advance; then, only a gate voltage less than the threshold voltage is needed to form a strong inversion layer, resulting in a smaller threshold voltage of the device [7].

**Figure 4:**
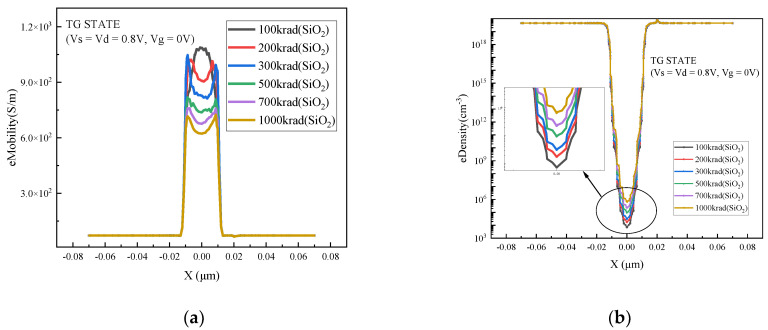
Electron density and mobility at the bottom of the channel: (**a**) mobility; (**b**) density.

**Figure 5:**
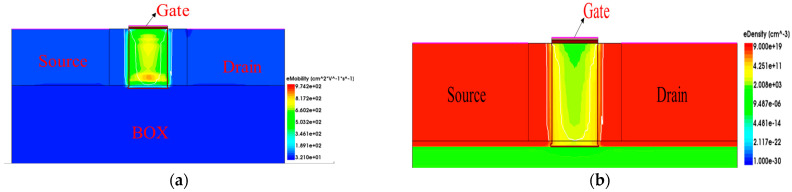
Two-dimensional cross-sectional diagram: (**a**) mobility; (**b**) density of fin.

Figure 4 and Figure 5 shows the electron density and mobility at the bottom of the channel at different radiation doses. As can be seen from Figure 4b, the higher the irradiation dose, the higher the concentration of positively charged trap charge in the buried oxygen layer; furthermore, the higher the concentration of converged electrons at the bottom of the channel, the greater the impact on the threshold voltage and off-state current. At the same time, as the collision probability between the carrier and the Si/SiO_2_ interface increases, the mobility of the electron surface decreases correspondingly.

#### 3.1.3. Influence of Temperature on TID

**Figure 6:**
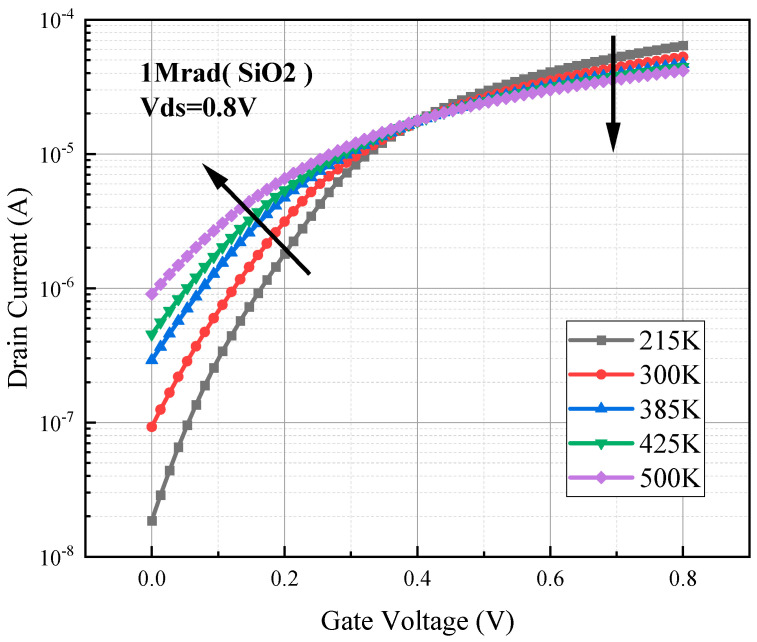
Degradation of transfer characteristic curves of irradiated 1000 krad (SiO_2_) dose devices at different temperatures.

Figure 6 shows the degradation of the transfer characteristic curves after simulating the total dose of 1 Mrad (SiO_2_) irradiation when the device is at 215 K, 300 K, 385 K, 425 K, and 500 K ambient temperatures. Since TCAD does not have a model that directly simulates the excitation of trap charges at high temperatures, we added a temperature-dependent SRH model to the TCAD software to indirectly simulate the excitation phenomenon of trap charges. Via this method, we obtained the same trend of the characteristic curves as that in [8], which proves that the method we used can more accurately simulate the influence of high temperature on the total dose effect. It is evident from Figure 6 that as the temperature increases, the five curves intersect at a zero-temperature coefficient (ZTC) point, where the drain current is temperature-independent when the device is operated at this point. The left end of the curve at the intersection point has positive drift and the right end has negative drift.

As the temperature increases, the device threshold voltage and off-state current degradation is more severe at the same irradiation dose. This is because the temperature increase intensifies the intrinsic excitation and increases the intrinsic carrier concentration in the channel, with a small change in concentration for the minority carriers (holes) in the channel, but an order of magnitude change in the majority carrier (electron) concentration. When the irradiation dose reaches 1000 krad (SiO_2_), a sufficient concentration of positively charged oxide trap charges accumulates in the BOX layer below the channel, and electrons converge in the channel under the attraction of these positive charges. The higher the temperature, the more intrinsic carriers in the channel and the more lessons (electrons) converge, creating a wider conducting channel, and thus exacerbating the degradation of threshold voltage and leakage current due to the total dose effect.

**Figure 7:**
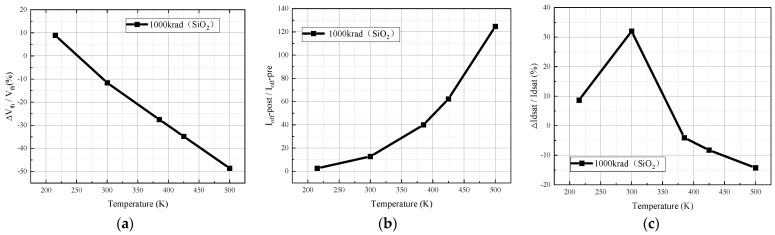
Degradation of device parameters for irradiated 1000 krad (SiO_2_) dose devices at different temperatures: (**a**) threshold voltage; (**b**) off-state current; (**c**) open-state current.

Another parameter that changes significantly due to temperature increase is the saturation drain current. As shown in Figure 7c, at different temperatures of an irradiation dose of 1000 krad (SiO_2_) in the simulation, the saturation drain current varies significantly; the saturation drain current increases with increasing temperature before the temperature reaches 300 K, and the opposite trend is observed after 300 K. 

The reasons for the changes in saturation leakage current are closely related to intrinsic excitation and changes in electron mobility [8]. The intrinsic excitation plays a major role before the temperature reaches 300 K. The increase in temperature exacerbates the intrinsic excitation, resulting in an increase in the concentration of oligons in the channel and a larger saturation drain current. The change in electron mobility after the temperature rise to 300 K becomes the main reason for the degradation of the saturation drain current. As the electron mobility becomes smaller, the saturation drain current during channel conduction becomes smaller as a result.

#### 3.1.4. Influence of Different Bias States on TID

Considering that there is no definite conclusion on which bias state is the worst bias state for irradiation of SOI FinFET devices, there is still a high necessity to simulate the total dose effect of the SOI FinFET and compare the degradation difference of the devices in different bias states.

TG state ((Vs = Vd = 0.8 V, Vg = 0 V)), OFF state (Vd = 0.8 V, vs. = Vg = 0 V), NULL state (Vd = vs. = Vg = 0 V), and ON state (Vg = 0.8 V, vs. = Vd = 0 V) are the four bias states that the device often exists in when it is exposed to radiation. With the irradiation dose rate set at 1 krad (SiO_2_)/s and the irradiation doses ranging from 100 krad (SiO_2_) to 1 Mrad (SiO_2_), the total dose effect simulations were carried out in the four distinct bias conditions. As we can see from Figure 6, the device’s transfer characteristic curves vary depending on whether it is in the TG, OFF, NULL, or ON bias states following simulated radiation. 

**Figure 8:**
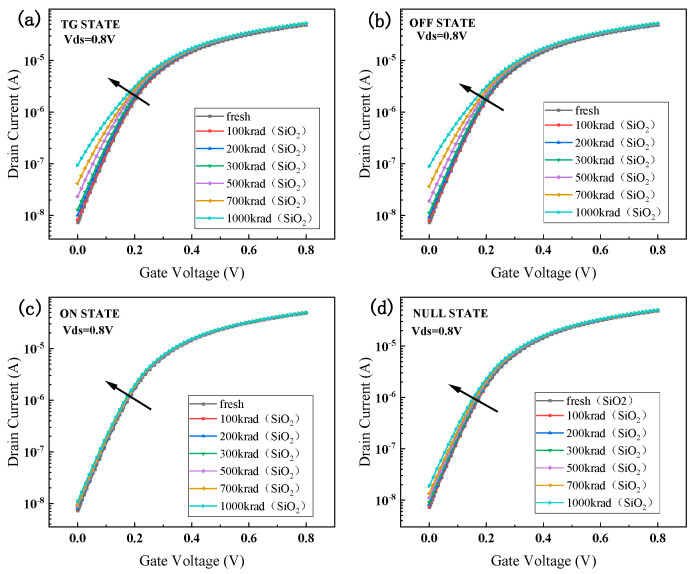
Transfer characteristic curves for different bias states: (**a**) TG state; (**b**) OFF state; (**c**) ON state; (**d**) NULL state.

From Figure 8, it can be observed that the TID affects the transfer characteristic curve of the device differently when the device is in different bias states. When the device is in the ON and NULL bias states, the TID on the device is relatively small, and the drift of the transfer characteristic curve is relatively low. However, when the device is in the OFF bias state or the TG bias state, the drift of the transfer characteristic curve is significantly higher. Regardless of the bias state in which the TID effect is simulated, the saturation drain current after channel conduction does not change much. However, there is a significant difference in the degree of off-state current degradation after irradiation of the device in different bias states. The degradation of the off-state current is highest in the TG and OFF bias states. Compared to the pre-irradiation device off-state current when the irradiation dose reaches 1000 krad (SiO_2_), the leakage current shows an order of magnitude jump. In contrast, when the device is biased in both the ON and NULL bias states, the off-state current varies less with dose and remains in the same order of magnitude before irradiation and up to 1000 krad (SiO_2_).

**Figure 9:**
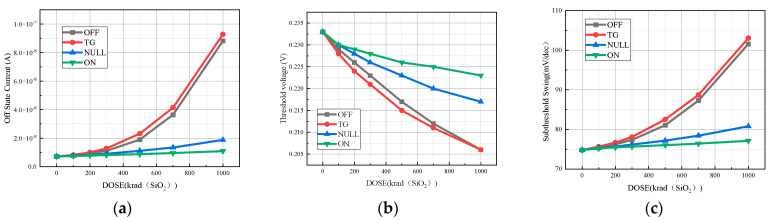
Degradation of electrical characteristic parameters in four bias states: (**a**) off-state current; (**b**) subthreshold swing; (**c**) threshold voltage.

As shown in Figure 9, when the device is in the TG state, the electrical parameters such as off-state current, threshold voltage, and subthreshold swing are degraded more severely with the accumulation of irradiation dose. When the irradiation dose reaches 1000 krad (SiO_2_), the off-state current of the device in both the transmission state and the off-state reaches 10 times that before irradiation, the threshold voltage degradation value is more than 10%, and the subthreshold swing degradation is more than 30%. When the device is in the ON and NULL bias states, the degradation of the device performance parameters due to the total dose effect is smaller. The off-state current is two times that before irradiation, the threshold voltage degradation value is less than 5%, and the subthreshold swing degradation is not more than 10%, which are all within the acceptable range. Therefore, it can be concluded from the simulation results in this section that for the 14 nm SOI FinFET device, the worst bias state is the transmission state, the optimal bias state is the OPEN state, and the OFF state is also a bias state that leads to serious degradation of the device performance.

**Figure 10:**
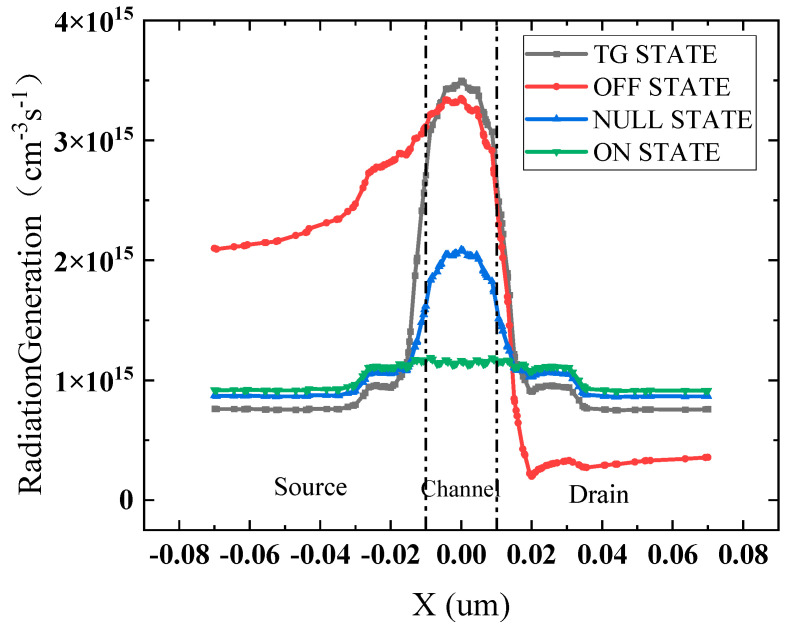
Irradiation-induced charge generation rates in the buried oxygen layer of devices in four different transport states at irradiation doses up to 1000 krad (SiO_2_).

From Figure 10, it is clear that the inductive charge generation rate is higher below the channel in the transfer and off-states, and when the device is in the OFF bias state, the source end also has a higher inductive charge generation rate. A positive voltage is applied to both the drain and the source of the device in the TG bias state, and the contact part of the drain–source region and the buried oxygen layer has a higher potential compared with other regions of the buried oxygen layer. Additionally, the irradiated inductive holes under the action of the electric field drift to the center and are captured by the hole traps in the buried oxygen layer below the channel, which results in a higher concentration of trap charges in the buried oxygen layer region below the channel of the device in the TG bias state and has a more serious impact on the electrical parameters of the device. Similarly, in the OFF bias state, only the drain terminal is connected to a high voltage. Under the action of the electric field, the positively charged oxide layer trap charge will also be gathered under the source region, causing the degradation of the device performance in the OFF bias state to be slightly weaker than in the TG bias state. Because, in the ON bias state, the gate is only connected to a high voltage, SOI FinFET devices, in addition to the top gate on both sides of the side gate, are in direct contact with the BOX layer. The two contact area potentials of 0.8 V, under the action of the electric field in the channel below the concentration of trap charges, are much smaller than in the other three bias states. As a result, the ON bias state of the device is affected by the minimum of the total dose effect, and the ON bias state is the optimal bias state.

### 3.2. HCI

#### 3.2.1. Mechanism and Physical Model of Action of HCI

With the continuous development of the semiconductor manufacturing process, the device channel is shortening, but the device’s normal operating voltage is not reducing by the same proportion. This leads to channel internal electric field intensity at a higher value, and the carriers become hot carriers under the electric field acceleration. Their own excess energy extends over the silicon oxide interface barrier into the silicon dioxide gate oxygen layer in the formation of the interfacial state, and the oxide layer traps the charge, affecting the device’s electrical characteristics [9].

There are several physical models in the simulation of HCI, such as the ionization collision model, the tunneling model, the channel hot carrier model, and carrier-carrier scattering. The hot carrier effect is an effect that decays with time; so, in the simulation process, we need to add an attenuation model to truly reflect this dynamic attenuation mechanism, so as to ensure the accuracy and reliability of the simulation results.

#### 3.2.2. Simulation of HCI

**Figure 11:**
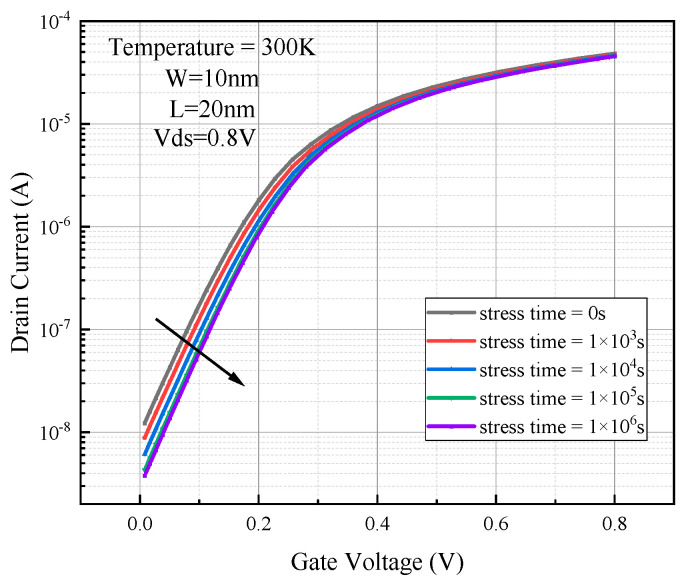
Comparison of transfer characteristic curve degradation of simulated hot carrier effect devices.

Based on the SOI FinFET device under the 14 nm process node model built in the previous section, the hot carrier effect was simulated under the hot carrier stress condition, with 1.2 V applied to the drain and the gate, and the source substrate was grounded. The simulation time was set to be 0 s, 10^3^ s, 10^4^ s, 10^5^ s, and 10^6^ s. The output transfer characteristic curves are shown at the end of the simulation in Figure 11.

The threshold voltage of the device increases and the saturated drain current and subthreshold swing decrease after simulating the hot carrier effect. When the hot carrier stress time reaches 10^6^ s, the threshold voltage degradation is over 15%, the subthreshold swing degradation is close to 3%, and the saturated drain current degradation is over 6% from Table 3.

As the hot carrier stress time becomes longer, the electrons in the channel near the drain side of the channel are accelerated by the electric field to obtain high energy. Thus, it becomes a hot carrier and is injected into the gate oxide layer by crossing the Si/SiO_2_ barrier under the action of the strong electric field. A portion of the hot electrons in the gate oxide flow out from the gate to become the gate current, and most of the remaining electrons are captured by the electron traps in the gate oxide to become the oxide trap charge, or reach the Si/SiO_2_ interface and form the interface state trap charge. The gate oxygen in the oxide layer trap charge and the Si/SiO_2_ interface at the interface state trap charge are negatively charged. Under the action of the Coulomb force, they repel the channel conductive electrons, equivalent to the gate adding a negative pressure. In order to counteract the Coulomb force of the oxide trap charge in the gate oxygen and the interfacial trap charge at the Si/SiO_2_ interface on the few electrons in the channel, it is necessary to give the gate a gate voltage greater than the threshold voltage in order to form a strong antipattern layer-conducting channel, so that the threshold voltage of the device will become larger under the action of the hot carrier effect. The electric field of the interface state trap charge itself is in the opposite direction to the gate electric field, and the two electric field effects cancel each other, reducing the ability of the gate voltage on the drain side of the channel to control the channel current, and thus the saturated drain current becomes gradually smaller [10].

#### 3.2.3. Influence of Temperature on HCI

**Figure 12:**
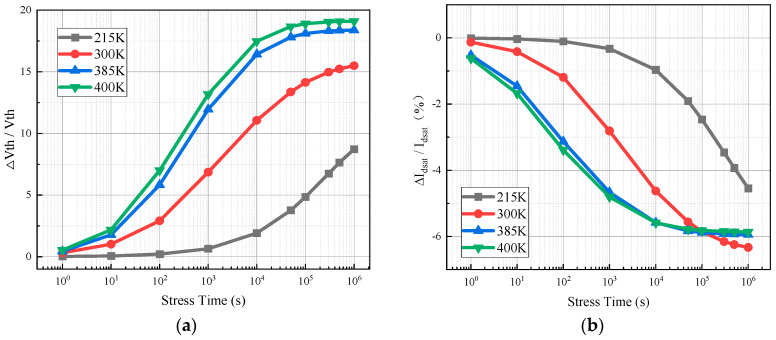
Parameter degradation of the simulated hot carrier effect device at different temperatures: (**a**) threshold voltage; (**b**) drain saturation current.

Figure 12 shows the degradation of the threshold voltage and saturated drain current of the device after simulating the applied hot carrier stress at 215 K, 300 K, 385 K, and 400 K ambient temperatures.

Figure 12 shows that the degradation rate of the saturation drain current and threshold voltage due to the hot carrier effect is higher in the device at high temperature. The higher the temperature, the more violent the thermal motion of the electrons and the higher their own energy; additionally, the carriers are more likely to have the energy to overcome the Si/SiO_2_ barrier. At the same time, high temperatures intensify intrinsic excitation and increase the concentration of intrinsic carriers in the channel, with a small change in concentration for the majority carriers (holes) in the channel, but an order of magnitude change in the concentration of the minority carriers (electrons). More electrons converge on the channel surface for the same gate voltage condition. Again, because of the increase in temperature, the lattice temperature inside the device rises, which has an effect on electron scattering in the channel. The channel is dominated by lattice scattering, and the lattice scattering is positively correlated with the temperature. As a result, the temperature increases, the scattering effect in the channel is stronger, the scattering probability is higher, and the carrier mean free range and mean free time are shortened. Gate voltage is fixed. The higher the internal temperature of the device, the higher the carrier (electron) concentration on the channel surface, the higher the kinetic energy, and the more serious the scattering. The more frequent the collision of the carriers in the surface channel movement process, the higher the collision ionization rate. Additionally, there will be more hot electrons at the interface between the channel and the gate medium, resulting in a more serious hot carrier effect [11]. When the device is in the low temperature state of 215 K, the electron concentration generated by the intrinsic excitation in the channel is very low, the kinetic energy of the electrons themselves is small, the number of hot carriers under the electric field acceleration is lower, and the number of interface state trap charges formed at the interface is smaller. Therefore, the device suffers less HCI damage at low temperature.

## 4. Coupling Effect of TID and HCI Simulation

### 4.1. HCI after TID

**Figure 13:**
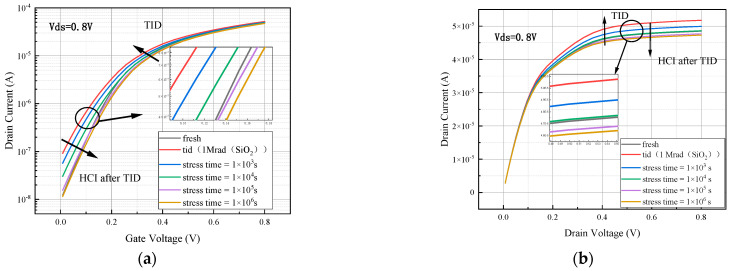
Degradation of device (**a**) transfer characteristic curve and (**b**) output characteristic curve after simulating TID and then HCI.

The total dose effect was first simulated in the transport state at an irradiation dose rate of 1 krad/s for 1000 s (1000 krad (SiO_2_)), and then the hot carrier effect was simulated under the hot carrier stress condition with a voltage of 1.2 V applied to the drain and the gate of the device. The source substrate was grounded, and the simulation times were set to 10^3^ s, 10^4^ s, 10^5^ s, and 10^6^ s. The output device transfer characteristic curve is shown in Figure 13.

**Figure 14:**
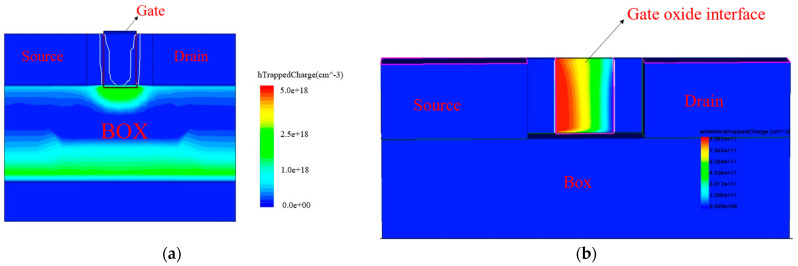
Trap charge inside the device: (**a**) trap charge in the buried oxygen layer at total irradiation dose of 1 Mrad/s(SiO_2_); (**b**) gate oxide interface state trap charge when stress time is 10^6^ s.

After the simulation of the total dose and hot carrier coupling effect, the device output characteristic curve changes are similar to the transfer characteristic curve changes. From Figure 13, it can be seen that the device output characteristic curve drifts upward after the simulation of irradiation with a total dose of 1 Mrad (SiO_2_). Because the total dose effect has less influence on the saturation drain current, the output characteristic curve drifts less after simulating the total dose effect. After that, the simulated hot carrier effect output characteristic curve first drifts back, and when the applied hot carrier stress reaches 10,000 s, the output characteristic curve has basically drifted back to the position before irradiation. It then continues to move in the negative direction toward the *Y*-axis until the stress time reaches 10^5^ s, and the curve drift begins to converge to a saturation value. From Figure 14, it can be seen that trap charge inside the device.

Due to the attraction of the trap charge in the buried oxygen layer to the channel electrons, a leakage channel is formed at the bottom of the channel, resulting in a decrease in the device threshold voltage and an increase in the off-state current. When the irradiation dose reaches 1 Mrad (SiO_2_), the threshold voltage of the device is degraded from 0.233 V to 0.206 V, and the off-state current increases from 7.2 × 10^−9^ A to 9.2 × 10^−8^ A. The change rate of the threshold voltage is more than ten percent, and the leakage current increases to thirteen times the original. However, under the action of the hot carrier effect, a large number of hot electrons are rapidly formed in the channel, and most of the hot carriers tunnel into the gate oxygen. There will also be a small number of hot carriers in the buried oxygen layer under the action of the Coulomb force of the trap charge that enter the buried oxygen layer and neutralize the oxide layer trap charge. The gate oxygen in the trap charge and the interface state are negatively charged, which will offset some of the buried oxygen layer of the positive oxide layer charge, and the total dose effect on the device is reduced by the hot carrier effect. The influence of the total dose effect on the device is offset by the hot carrier effect; the threshold voltage begins to drift positively and the drain current gradually decreases. Table 4 shows that the threshold voltage returns to the initial value before irradiation after applying hot carrier stress for more than 10,000 s, and the threshold voltage continues to increase after continuing to apply hot carrier stress. After applying hot carrier stress for 10^6^ s, the threshold voltage starts to converge to a limiting value, which is less than five percent larger than that before simulating the coupling effect; the off-state current decreases to 1.0 × 10^−8^ A, which is not yet restored to the initial value before irradiation, but is already in the same order of magnitude.

When the device is irradiated, a positively charged oxide trap charge is generated in the oxide layer. Under the action of the Coulomb force, the carrier (electron) in the channel is attracted and converges to the lower surface of the channel, forming a weak conductive channel, causing the device to still have a large leakage current when the gate voltage is zero. Then, only a gate voltage less than the threshold voltage can form a strong inverse layer in the channel. This also explains the effect of the oxide trap charge on threshold voltage and leakage current. When the irradiated device is under hot carrier stress, the radiation-generated charge in the buried oxide layer is neutralized by a small number of injected hot electrons in the channel, which restores the drift in parameters such as the threshold voltage due to the total dose effect [12]. At the same time, some hot electrons in the channel cross the Si/SiO_2_ barrier and form an interface state trap charge at the interface between the channel and the gate oxide layer. The negatively charged interface state will prevent the carrier (electron) in the channel from converging to form a conductive channel. Therefore, a voltage greater than the threshold voltage needs to be applied to the gate to offset the effect of the interface state and form a strong inverse layer [13]. This also explains the effect of interface trap charges on threshold voltage.

### 4.2. TID after HCI

The hot carrier effect was first simulated under the stress condition of 1.2 V applied to the gate–drain and the source–substrate ground for 10^6^ s. After that, the device was put in the transport state to simulate the total dose effect at an irradiation dose rate of 1 krad/s, and the simulation times were set to 100 s (100 krad (SiO_2_)), 500 s (500 krad (SiO_2_)), and 1000 s (1000 krad (SiO_2_)). After the simulation, the drain voltage was fixed at 0.8 V, the source and the substrate were grounded, and the gate voltage was swept from 0 to 0.8 V in the output transfer characteristic curve.

**Figure 15:**
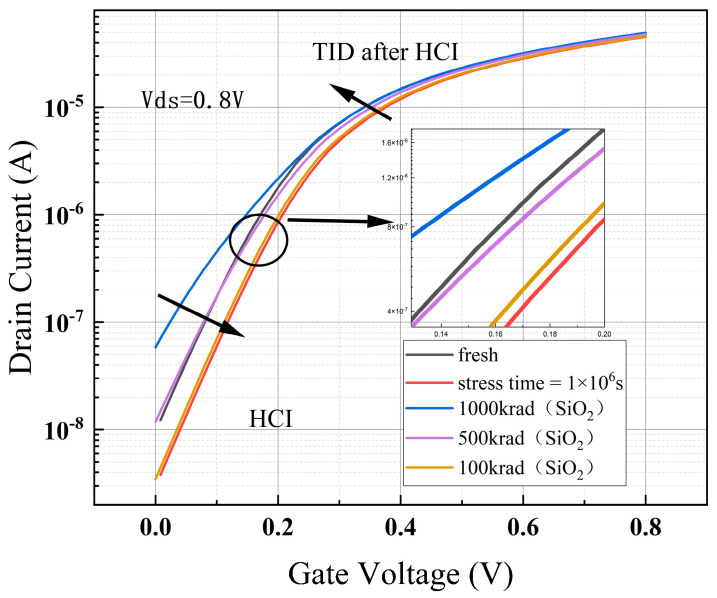
Degradation of device transfer characteristic curve after simulating TID and then HCI.

From Figure 15, it can be seen that in the device simulation of the applied hot carrier stress, 10^6^ s after the simulation of the total dose effect, the transfer characteristic curve first drifted in the positive direction of the *Y*-axis, and then drifted back. At the irradiation time of about 500 s, the transfer characteristic curve drifted back to the position of the simulation of the hot carrier effect before the position. Continuing irradiation, the transfer characteristic curve also moved in the negative direction of the *Y*-axis until the curve drifted towards a saturation value. When the irradiation time reached 500 s, the device off-state current returned to the same order of magnitude as before the simulated HCI, but the curvature of the transfer characteristic curve was smaller than before the simulated HCI, i.e., the subthreshold amplitude was larger.

The hot carrier effect on the electrical characteristics of the device is mainly reflected in the two parameters of threshold voltage and saturated drain current, as can be seen from Table 5. After the application of 10^6^ s of hot carrier stress, the device threshold voltage increased by fifteen percent and the saturated drain current decreased by six percent. After the simulation of an irradiation dose of 1 Mrad (SiO_2_), the threshold voltage was still higher than the initial value of the device, by two percent, but the saturated drain voltage reversed from negative to positive by two percentage points. The off-state current and subthreshold swing were not sensitive to the hot carrier effect, as the off-state current was halved and the subthreshold swing was reduced by only two percentage points after 10^6^ s of hot carrier stress, both of which are relatively small changes. Afterwards, the simulation of an irradiation dose of 1 Mrad (SiO_2_) dramatically increased the values of both parameters; the off-state current increased by orders of magnitude to 8.2 times the initial value, and the subthreshold amplitude of the pendulum increased by thirty percent compared with the initial value.

The hot carrier effect on the electrical characteristics of the device is mainly reflected in the two parameters of threshold voltage and saturated drain current, as can be seen from Table 5. After the application of 10^6^ s of hot carrier stress, the device threshold voltage increased by fifteen percent and the saturated drain current decreased by six percent. After the simulation of an irradiation dose of 1 Mrad (SiO_2_), the threshold voltage was still higher than the initial value of the device by two percent, but the saturated drain voltage reversed from negative to positive by two percentage points. The off-state current and subthreshold swing were not sensitive to the hot carrier effect, as the off-state current was halved and the subthreshold swing was reduced by only two percentage points after 10^6^ s of hot carrier stress, both of which are relatively small changes. Afterwards, the simulation of an irradiation dose of 1 Mrad (SiO_2_) dramatically increased the values of both parameters; the off-state current increased by orders of magnitude to 8.2 times the initial value, and the subthreshold amplitude of the pendulum increased by thirty percent compared with the initial value.

Whether the total dose effect is simulated first and then the hot carrier effect is simulated, or the hot carrier effect is simulated first and then the total dose effect is simulated, these two coupling simulation methods will make the effects of TID and HCI on the device cancel each other out. The Table 6 shows the degradation of electrical characteristics of the device under different coupling methods for the simulation of a 1000 krad (SiO_2_) irradiation dose and 10^6^ s of hot carrier stress. It can be seen that the difference lies in the threshold voltage and saturated drain current changes, which are smaller after the first HCI TID, and the threshold voltage under this coupling method also experiences positive growth; however, the growth is less than three percent. Saturated drain current experiences positive growth and the value of change is less than 2.5 percent. With TID first, followed by HCI, the off-state current and subthreshold swing changes are even smaller; the growth in off-state current under this coupling method is only 1.4%, and sub-threshold swing growth is 20%.

As the duration of HCI and TID action increases, the positively charged oxide trap charge induced by the total dose effect promotes channel carrier formation, whereas there is a negatively charged interfacial state induced by the hot carriers that hinders channel carrier formation. Thus, it is the reciprocal compensation between the two effects that leads to the mutual cancellation of the two effects.

### 4.3. Influence of Different Bias States on Coupling Effect

The hot carrier effect was first simulated under the stress condition of 1.2 V applied to the gate–drain and the source–substrate ground for 10^6^ s. After that, the device was put in TG state, OFF state, N state, and NULL state to simulate the total dose effect at an irradiation dose rate of 1 krad/s, and the simulation time was set to 1000 s (1000 krad (SiO_2_)). After the simulation, the drain voltage was fixed at 0.8 V, the source and the substrate were grounded, and the gate was swept from 0 to 0.8 v in the output transfer characteristic curve.

**Figure 16:**
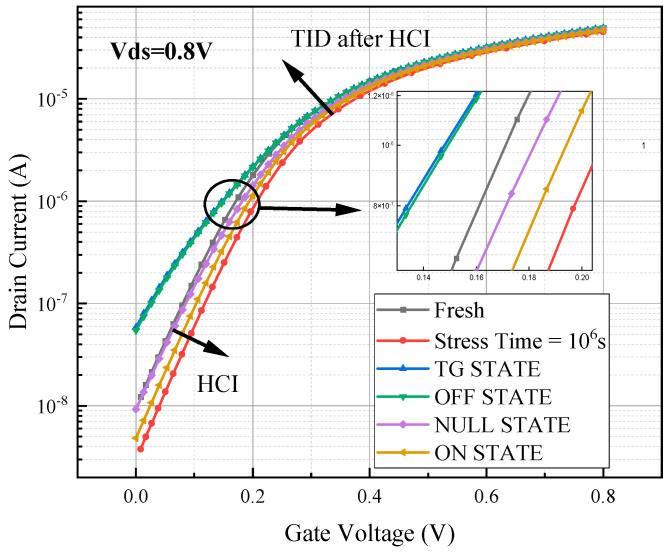
Degradation of the device transfer characteristic curve of the simulated coupling effect under different bias states.

From Figure 16, it can be seen that regardless of the bias state of the device, the total dose effect can offset the influence of the hot carrier effect for the device after simulating HCI and then TID. When the device was put in the OFF state and TG state, the curve drifted back to a greater degree, followed by the NULL state, and the drift degree of the transfer characteristic curve was the least when the device was in the ON state. Compared with the initial transfer characteristic curve of the device, it can be seen that the damage of TID exceeds that of HCI when the device is irradiated for 1000 s in the OFF state and TG state.

Regardless of the bias state of the device, the change in the device threshold voltage shows a tendency of increasing and then decreasing after simulating HCI and then TID, as can be seen from Table 7. When the hot carrier stress is applied for 106 s, the threshold voltage increases from the initial value of 0.233 V to 0.268 V. According to the JEDEC standard, the device is judged to have failed when the degradation of the electrical parameters exceeds 10%. In this case, the device threshold voltage increased by fifteen percent, indicating that the device failed. Subsequent irradiation simulations were performed, and the threshold voltage increase fell back to within 10% in all bias states, except the ON state, and the device returned to the non-failed state.

The Table 8 shows the variation in the device off-state current and the drain saturation current, and it can be seen that the device off-state current and the drain saturation current show a tendency of decreasing and then increasing. The drain saturation current changes for the simulated coupling effect are generally small for the four bias states, with degradation in the plus or minus 5% range. In contrast to the drain saturation current, the off-state current varies more significantly, increasing more than sevenfold in both bias states, i.e., the OFF state and the TG state. The off-state currents degrade due to the coupling effect being dominated by TID.

**Figure 17:**
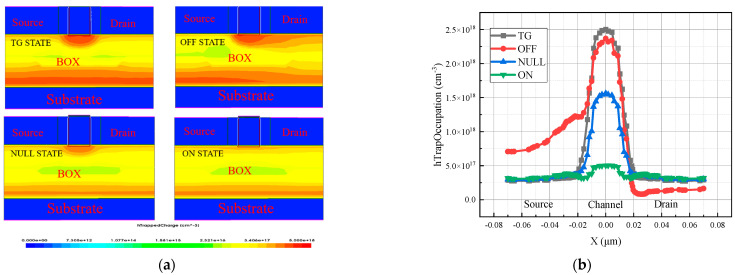
(**a**) Trap charge inside the device in the buried oxygen layer; (**b**) trap charge concentration in the buried oxygen layer under different bias states.

From Figure 17a, trap charges are mainly distributed in the buried oxygen layer below the channel when the device simulates the coupling effect in the TG state, ON state, and NULL state. When the device is in the OFF state, the trap charge is mainly distributed in the buried oxygen layer below the channel, in addition to some trap charge being distributed below the source region. From the data in Figure 17b, it can be observed that the oxide trap charge concentration in the buried oxygen layer is significantly higher when the device is in the TG and OFF states, and the transfer characteristic curves drift backward to a higher degree. The NULL state is followed by a lower concentration of oxide trap charge in the buried oxygen layer when the device is in the ON state, and the curve drifts backward to the lowest degree.

## 5. Conclusions

In this study, TID, HCI, and the coupling effect of TID and HCI on SOI FinFET devices were investigated. The simulation results show that TID causes the threshold voltage of the device to decrease, and the subthreshold swing and off-state current to increase. When the irradiation dose reaches 1 Mrad (SiO_2_), the threshold voltage degradation is more than 10%, the subthreshold swing degradation is nearly 40%, and the off-state current increases to 13 times the initial value. When irradiated with 1 Mrad (SiO_2_) at low temperature, the influence of temperature on the threshold voltage of the device exceeds that of TID, and the threshold voltage value drifts by nearly 10%. When the simulated irradiation of 1 Mrad (SiO_2_) was carried out at a high temperature (500 K), the on-state current of the device also degraded by nearly 15%. The effect of HCI on the device is mainly reflected in the threshold voltage and saturation drain current. When the hot carrier stress time reaches 10^6^ s, the threshold voltage degradation is more than 15% and the saturation drain current degradation is more than 6%. The HCI of the device becomes more severe as the temperature increases. When the simulations of TID and HCI are coupled, the damages of the two effects on the device offset each other. If HCI is simulated first and then TID, the degradation of the subthreshold swing and the off-state current is more severe; if TID is simulated first and then HCI, the degradation of the threshold voltage and the saturation drain current is more severe. However, as long as TID and HCI work together, the device will have a longer service life.

## Figures and Tables

**Table 1:** Device parameters used for simulation of SOI FinFET devices.

Parameter	Data
Gate length (nm)	20
Channel width (nm)	10
Channel length (nm)	26
Gate oxide thickness (nm)	0.9
Buried oxide thickness (T_box_, nm)	50
Buried oxide length (nm)	140
Source/drain extension length (nm)	10
Source/drain length (nm)	50
Source/drain width (nm)	40
Channel doping (cm^−3^)	1.0 × 10^15^
Source/drain doping (cm^−3^)	4.5 × 10^19^
Gate work function (eV)	4.38

**Table 2:** Simulation of TID device parameter degradation.

Dose (krad (SiO_2_))	∆V_th_/V_th_ (%)	I_off_-Post/I_off_-Pre	∆SS/SS (%)
0	0	1	0
100	−1.65	1.13	1.15
200	−3.73	1.38	2.51
300	−5.08	1.77	4.41
500	−7.51	3.19	10.3
700	−9.35	5.71	18.6
1000	−11.63	12.78	37.7

**Table 3:** Simulation of HCI device parameter degradation.

Stress Time (s)	∆V_th_/V_th_ (%)	I_off_-Post/I_off_-Pre	∆SS/SS (%)
0	0	0	0
10^3^	6.66	−2.81	−0.13
10^4^	10.74	−4.62	−0.40
10^5^	13.74	−5.85	−2.27
10^6^	15.07	−6.33	−2.67

**Table 4:** Degradation of the electrical characteristics of the device after simulating TID and then HCI.

	∆V_th_/V_th_ (%)	I_off_-Post/I_off_-Pre	∆SS/SS (%)	∆I_dsat_/I_dsat_ (%)
Fresh	0	1	0	0
TID (1000 krad (SiO_2_))	−11.6	12.8	37.7	8.9
HCI (1000 s)	−7.7	7.9	33.6	2.9
HCI (10^4^ s)	−1.2	4.2	28.5	0.2
HCI (10^5^ s)	3	2.1	22.9	−1.7
HCI (10^6^ s)	4.3	1.4	20.0	−2.5

**Table 5:** Degradation of the electrical characteristics of the device after simulating HCI and then TID.

	∆V_th_/V_th_ (%)	I_off_-Post/I_off_-Pre	∆SS/SS (%)	∆Idsat/Idsat (%)
Fresh	0	1	0	0
HCI (10^6^ s)	15	0.47	−2.7	−6.2
TID (100 krad (SiO_2_))	14.2	0.48	−2.1	−5.4
TID (500 krad (SiO_2_))	7.3	1.64	6.9	−1
TID (1000 krad (SiO_2_))	2.6	8.2	31.5	2.1

**Table 6:** Degradation of electrical characteristic parameters of devices with different coupling simulation methods.

	∆V_th_/V_th_ (%)	∆I_dsat_/I_dsat_ (%)	I_off_-Post/I_off_-Pre	∆SS/SS (%)
HCI after TID	4.3	−2.5	1.4	20
TID after HCI	2.6	2.1	8.2	31.5

**Table 7:** The change in the device threshold voltage of the simulated coupling effect under different bias states.

	Threshold Voltage (V)	∆Vth/Vth (%)
Fresh	0.233	0.0
HCI (10^6^ s)	0.268	15.0
TID (OFF)	0.240	3.0
TID (TG)	0.239	2.6
TID (ON)	0.259	11.1
TID (NULL)	0.252	8.1

**Table 8:** The change in device drain saturation current and off-state current of the simulated coupling effect under different bias states.

	Drain Saturation Current (A)	∆I_dsat_/I_dsat_ (%)	Off-State Current (A)	I_off_-Post/ I_off_-Pre
Fresh	4.85 × 10^−5^	0.0	7.20 × 10^−9^	1.0
HCI (10^6^ s)	4.54 × 10^−5^	−6.2	3.40 × 10^−9^	0.5
TID (OFF)	4.95 × 10^−5^	2.1	5.44 × 10^−8^	7.6
TID (TG)	4.94 × 10^−5^	2.1	5.85 × 10^−8^	8.2
TID (ON)	4.68 × 10^−5^	−3.5	4.83 × 10^−9^	0.7
TID (NULL)	4.77 × 10^−5^	−1.6	9.26 × 10^−9^	1.3

## Data Availability

The original contributions presented in the study are included in the article, further inquiries can be directed to the corresponding author.
